# Radiofrequency bipolar hemostatic sealer reduces blood loss, transfusion requirements, and cost for patients undergoing multilevel spinal fusion surgery: a case control study

**DOI:** 10.1186/s13018-014-0050-2

**Published:** 2014-07-05

**Authors:** Steven M Frank, Jack O Wasey, Ian M Dwyer, Ziya L Gokaslan, Paul M Ness, Khaled M Kebaish

**Affiliations:** 1 Department of Anesthesiology/Critical Care Medicine, The Johns Hopkins Medical Institutions, Zayed 6208, 1800 Orleans Street, Baltimore 21287, MD, USA; 2 Department of Neurosurgery, Oncology, and Orthopaedic Surgery, The Johns Hopkins Medical Institutions, Zayed 6208, 1800 Orleans Street, Baltimore 21287, MD, USA; 3 Department of Pathology, The Johns Hopkins Medical Institutions, Zayed 6208, 1800 Orleans Street, Baltimore 21287, MD, USA; 4 Department of Orthopaedic Surgery, The Johns Hopkins Medical Institutions, Zayed 6208, 1800 Orleans Street, Baltimore 21287, MD, USA; 5 Patient Blood Management Program, Department of Anesthesiology/Critical Care Medicine, The Johns Hopkins Medical Institutions, Zayed 6208, 1800 Orleans Street, Baltimore 21287, MD, USA

**Keywords:** Bipolar, Blood, Cautery, Hemostasis, Radiofrequency, Sealer

## Abstract

**Background:**

A relatively new method of electrocautery, the radiofrequency bipolar hemostatic sealer (RBHS), uses saline-cooled delivery of energy, which seals blood vessels rather than burning them. We assessed the benefits of RBHS as a blood conservation strategy in adult patients undergoing multilevel spinal fusion surgery.

**Methods:**

In a retrospective cohort study, we compared blood utilization in 36 patients undergoing multilevel spinal fusion surgery with RBHS (Aquamantys®, Medtronic, Minneapolis, MN, USA) to that of a historical control group (*n* = 38) matched for variables related to blood loss. Transfusion-related costs were calculated by two methods.

**Results:**

Patient characteristics in the two groups were similar. Intraoperatively, blood loss was 55% less in the RBHS group than in the control group (810 ± 530 vs. 1,800 ± 1,600 mL; *p* = 0.002), and over the entire hospital stay, red cell utilization was 51% less (2.4 ± 3.4 vs. 4.9 ± 4.5 units/patient; *p* = 0.01) and plasma use was 56% less (1.1 ± 2.4 vs. 2.5 ± 3.4 units/patient; *p* = 0.03) in the RBHS group. Platelet use was 0.1 ± 0.5 and 0.3 ± 0.6 units/patient in the RBHS and control groups, respectively (*p* = 0.07). The perioperative decrease in hemoglobin was less in the RBHS group than in the control group (−2.0 ± 2.2 vs. –3.2 ± 2.1 g/dL; *p* = 0.04), and hemoglobin at discharge was higher in the RBHS group (10.5 ± 1.4 vs. 9.7 ± 0.9 g/dL; *p* = 0.01). The estimated transfusion-related cost savings were $745/case by acquisition cost and approximately 3- to 5-fold this amount by activity-based cost.

**Conclusions:**

The use of RBHS in patients undergoing multilevel spine fusion surgery can conserve blood, promote higher hemoglobin levels, and reduce transfusion-related costs.

## Background

Given the recognized risks [[1]–[3]] and expense [[4],[5]] of allogeneic blood transfusion in surgical patients, a successful blood management program should include efforts to reduce intraoperative blood loss as a primary measure of blood conservation. Patients undergoing posterior spinal fusion surgery can lose substantial amounts of blood, which can prolong operative time and obscure visualization of important neural structures. Such patients often require transfusion of allogeneic blood components [[6]]. Methods to reduce transfusion requirements during spine surgery include controlled hypotension [[7]], autologous blood salvage [[8],[9]], and antifibrinolytic medications [[10]]. Another method that has been described recently is a relatively new electrocautery device, which can be described as a saline-irrigated radiofrequency bipolar hemostatic sealer (RBHS). Unlike conventional electrocautery, which burns small blood vessels at temperatures as high as 400°C, RBHS seals blood vessels by delivering water-cooled energy that causes vascular contraction at a tissue temperature <100°C [[11],[12]].

Studies have shown that bleeding is reduced when RBHS is used during liver resection [[13]], total hip arthroplasty [[14]], and total knee arthroplasty [[15]]. To our knowledge, only one study has evaluated the utility of RBHS in complex spine surgery. In that study, Mankin et al. [[16]] reported significant reductions in blood loss (57%) and transfusion rates (from 6.6% to 0%) in adolescent patients undergoing surgery for scoliosis; however, they gave only the percentage of patients transfused without reporting the amount of blood transfused or any hemoglobin data. In the current study, we compared blood loss and transfusion requirements in adults undergoing multilevel spinal fusion surgery with RBHS to those of a similar patient cohort in which only conventional unipolar cautery was used. Our blood management program maintains two large databases that accurately monitor both intraoperative and perioperative (entire hospital stay) transfusion of all blood components, including red blood cells (RBCs), fresh frozen plasma (FFP), and platelets (PLTs). Using these databases, we assessed blood loss, transfusion requirements, and estimated transfusion-related costs to assess the utility of RBHS in adult patients undergoing multilevel spinal fusion surgery.

## Methods

After receiving approval from the Johns Hopkins Hospital institutional review board, we performed a retrospective cohort study, whereby 36 consecutive patients undergoing multilevel spinal fusion surgery with the RBHS device (Aquamantys®, Medtronic, Minneapolis, MN, USA) were compared to a historical control group (*n* = 38) that was propensity-matched for important variables related to blood loss. These variables included the number of spinal levels fused, type of surgical procedure (lumbar, thoracic, or thoracolumbar), the nine specific surgeons performing the procedures, and the percentage of patients undergoing revision spinal surgery. Patients in the matched control group were selected from the operating room medical information system (ORMIS) database and underwent surgery between August 2012 and January 2013. Patients in the RBHS group were selected as consecutive surgical cases during July and August of 2013, when the RBHS was introduced. Exclusion criteria were preoperative coagulopathy, thrombocytopenia, and refusal of blood product transfusion.

The RBHS device was set to a power setting of 140, and the saline flow rate was set to medium. Traditional unipolar cautery (ConMed System 5000™, Utica, NY, USA) and intraoperative autologous blood salvage (Brat-2, Sorin Group, Milan, Italy) were used in both study groups. An adult-size 225-mL bowl was used for blood salvage, and salvaged blood was processed and transfused if the collected volume exceeded 500 mL. Intraoperative hemoglobin (Hb) triggers were used according to the usual practice at our institution for this type of surgery (9–10 g/dL intraoperatively and 8–9 g/dL postoperatively). FFP and PLTs were transfused according to the clinical judgment of the anesthesiologists and surgeons. Typically, FFP and PLTs were given if the transfusion requirements exceeded ½ of the patient's total blood volume (≈5 units RBCs).

Blood utilization data were obtained from two separate databases. Intraoperative data were obtained from the electronic anesthesia records (Metavision®, iMdSoft, Needham, MA, USA). A detailed description of this database and its validation have been published previously [[6]]. Whole hospitalization blood utilization data and hemoglobin data (from admission to discharge) were obtained from a blood management intelligence portal, IMPACT Online® (Haemonetics, Braintree, MA, USA) [[17]]. This database also includes morbidity outcomes based on ICD-9 codes assigned to each patient upon discharge. We compared the following morbidity outcomes between the two groups: infection, thrombotic, renal, respiratory, stroke, and myocardial infarction. We also analyzed a composite outcome that was the presence of any of these morbid events.

We calculated transfusion-related cost by two different methods. The first method is based on the average cost to acquire blood components from blood suppliers in the mid-Atlantic region (acquisition cost). These costs were calculated as $220/RBC unit, $50/FFP unit, and $600/PLT unit. The second method is the activity-based cost of blood, as described by Shander et al. [[4]], which accounts for every step in the process of bringing blood from the donor to the recipient, including all transfusion-related activities, such as collection, transport, testing, storage, administrative work, and the transfusion process itself. This cost was determined to be between 3.2 and 4.8 times the acquisition cost. The average incremental equipment cost was $493 per surgical case when the RBHS was used.

### Sample size and statistical analysis

We performed a sample size calculation based on the known blood utilization for these types of cases at our institution [[6]]. To detect a 25% difference in the number of blood product units transfused with an *α* of 0.05 and power of 0.8, we required an estimated sample size of 32 patients per study group.

Differences in the mean measured values between groups were analyzed by Student's *t* tests. Dichotomous variables were analyzed by the chi-squared test. All tests were two-tailed. All data are presented as mean ± SD, and *p* < 0.05 was used to define significance.

## Results

### Preoperative and intraoperative patient characteristics

The RBHS and control groups were similar for the variables of age, sex, body mass, number of spinal levels fused, and preoperative hemoglobin (Table 1). The percentages of patients undergoing thoracic, lumbar, and thoracolumbar fusion and the percentage of patients undergoing revision surgery were also similar in the two groups. Surgeon specialty (neurosurgery/orthopedic surgery) and duration of surgery were similar between groups.

**Table 1:** Comparison of the radiofrequency bipolar hemostatic sealer and control groups

**Variable**	**RBHS group (*n*** **= 36)**	**Control group (*n*** **= 38)**	***P*** **value**
Age (years)	64 ± 14	59 ± 13	0.17
Sex (male/female)	18/18	23/15	0.33
Body mass (kg)	76.6 ± 18.4	74.1 ± 21.2	0.62
Spinal levels fused (median (IQR))	4 (3, 9)	4 (2, 7)	0.23
Spinal levels fused (mean ± SD)	6.1 ± 4.5	4.9 ± 3.7	0.22
Thoracic fusion	4 (11.1%)	4 (10.5%)	0.67
Lumbar fusion	18 (50%)	20(52.6%)	0.56
Thoracolumbar fusion	14 (38.9%)	14 (36.9%)	0.72
Surgeon specialty (neuro/ortho)	12/24	12/26	0.52
Duration of surgery (h)	6.07 ± 0.42	6.14 ± 0.37	0.89
Revision surgery	8 (22.2%)	7 (18.4%)	0.78
ASA class	2.7 ± 0.1	2.5 ± 0.1	0.19

*ASA* American Society of Anesthesiologists, *IQR* interquartile range, *RBHS* radiofrequency bipolar hemostatic sealer, *SD* standard deviation, *Neuro* neurosurgery, *Ortho* orthopedic surgery.

### Blood loss and hemoglobin levels

**Figure 1:**
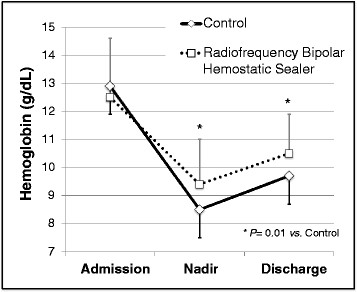
**Average hemoglobin concentrations compared in the radiofrequency bipolar hemostatic sealer (RBHS) and control groups.** Baseline hemoglobin was similar between groups, but the decrease in hemoglobin was less in the RBHS group (*P* = 0.04), and the hemoglobin at discharge was higher in the RBHS group (*P* = 0.01).

Intraoperatively, estimated blood loss was 55% less in the RBHS group than in the control group (*p* = 0.002). Likewise, the volume of colloid used was 63% less (*p* = 0.04), and the volume of returned salvaged blood was 54% less (*p* = 0.003) in the RBHS group than that in the control group (Table 2). The nadir hemoglobin during hospitalization and the hemoglobin upon discharge were higher in the RBHS group (Table 2). The decrease in hemoglobin from admission to discharge was 1.2 g/dL less in the RBHS group than that in the control group (*p* = 0.04), and the hemoglobin concentration at discharge was 0.8 g/dL higher in the RBHS group (*p* = 0.01) than that in the control group. These changes in hemoglobin levels are summarized in Figure 1.

**Table 2:** Comparison of the radiofrequency bipolar hemostatic sealer and control groups

**Variable**	**RBHS group (*n*** **= 36)**	**Control group (*n*** **= 38)**	***P*** **value**
Hemoglobin, preoperative (g/dL)	12.5 ± 2.1	12.9 ± 2.1	0.47
Hemoglobin, hospital nadir (g/dL)	9.4 ± 1.6	8.5 ± 1.1	0.01
Hemoglobin upon discharge (g/dL)	10.5 ± 1.4	9.7 ± 0.9	0.01
Δ Hemoglobin (admit - discharge) (g/dL)	−2.0 ± 2.2	−3.2 ± 2.1	0.04
Crystalloid (mL)	6,000 ± 2,000	5,900 ± 2,300	0.88
Colloid volume (mL)	70 ± 170	190 ± 270	0.04
Cell salvage (volume returned (mL))	230 ± 47	500 ± 200	0.003
Estimated blood loss (mL)	810 ± 530	1,800 ± 1,600	0.002

*RBHS* radiofrequency bipolar hemostatic sealer.

### Transfusion requirements

**Figure 2:**
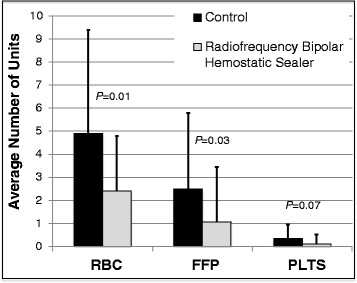
**Blood utilization (from admission to discharge) compared in the radiofrequency bipolar hemostatic sealer (RBHS) and control groups.** Compared to the control group, the RBHS group used 51% fewer units of red blood cells (*P* = 0.01) and 56% fewer units of fresh frozen plasma (*P* = 0.03). The RBHS group also used fewer platelets (PLTS), but the difference from control did not reach statistical significance (*P* = 0.07).

We also compared the transfusion requirements in the two groups (Table 3). Intraoperatively, the RBHS group required 57% fewer units of RBCs (*p* = 0.009) and 83% fewer units of FFP (*p* = 0.002) than did the control group. The two groups did not differ significantly in PLT requirements. Over the entire hospital stay, the RBHS group received 51% fewer units of RBCs (*p* = 0.01) and 56% fewer units of FFP (*p* = 0.03) than did the control group (Figure 2). Although the RBHS group received fewer platelets than the control group did, the difference was not statistically significant (*p* = 0.07).

**Table 3:** Intraoperative and whole hospitalization transfusion requirements

**Variable**	**RBHS group (*n*** **= 36)**	**Control group (*n*** **= 38)**	***P*** **value**
Transfusion (intraoperative)			
RBC (units/patient)	1.2 ± 1.4	2.8 ± 3.4	0.009
FFP (units/patient)	0.3 ± 0.7	1.8 ± 2.8	0.002
PLTs (units/patient)	0 ± 0	0.1 ± 0.4	0.11
RBCs given	19 (53%)	24 (63%)	0.48
FFP given	7 (19%)	16 (42%)	0.03
PLTs given	0 (0%)	3 (8%)	0.24
Transfusion (whole hospital stay)			
RBC (units/patient)	2.4 ± 2.4	4.9 ± 4.5	0.01
FFP (units/patient)	1.1 ± 2.4	2.5 ± 3.4	0.03
PLTs (units/patient)	0.1 ± 0.5	0.3 ± 0.6	0.07
RBCs given	21 (58%)	30 (79%)	0.05
FFP given	7 (19%)	6 (42%)	0.04
PLTs given	0 (0%)	3 (8%)	0.24

*FFP* fresh frozen plasma, *PLTs* platelets, *RBC* red blood cells, *RBHS* radiofrequency bipolar hemostatic sealer.

### Outcome data

The incidence of morbid events and the composite outcome of any morbid event were similar in the two groups (Table 4). The difference in length of stay was not statistically significant (6.8 ± 4.1 days in the RBHS group and 8.1 ± 6.4 days in the control group; *p* = 0.35).

**Table 4:** Comparison of clinical outcomes in the radiofrequency bipolar hemostatic sealer and control groups

**Variable**	**RBHS group (*n*** **= 36)**	**Control group (*n*** **= 38)**	***P*** **value**
Composite outcome (any morbid event)	1 (2.8)	5 (13.1)	0.20
Morbid events^a^			
Infection	0 (0%)	1 (2.8%)	0.20
Thrombotic	1 (2.8%)	2 (5.3%)	0.87
Renal	1 (2.8%)	2 (5.3%)	0.87
Respiratory	0 (0%)	0 (0%)	NS
Stroke	0 (0%)	0 (0%)	NS
Myocardial infarction	0 (0%)	0 (0%)	NS

*NS* not significant, *RBHS* radiofrequency bipolar hemostatic sealer. ^a^Morbid events were determined by ICD-9 codes at patient discharge from IMPACT Online (Haemonetics Corp, Braintree, MA, USA).

### Cost savings

When we multiplied the acquisition costs for each unit of blood component in US dollars by the number of units saved (difference between groups) during the entire hospital stay, we found that use of the RBHS device saved an average blood cost of $745/patient (Table 5). Using the activity-based blood costs [[4]], the cost savings attributable to the RBHS were between $2,384 and $3,576/patient. After accounting for the RBHS device cost, the net cost savings were $252/patient (using the blood acquisition cost), and $1,891–$3,083/patient (using the activity-based cost). This however, does not include any incremental costs of evaluating and/or treating transfusion-related complications.

**Table 5:** Units of blood product saved and costs saved per surgical case

**Blood component**	**Units**	**Acquisition cost**^**a**^	**Activity-based cost**^**b**^
Savings in RBCs	2.5	$550	$1,760–$2,640
Savings in FFP	1.4	$75	$240–$360
Savings in PLTs	0.2	$120	$384–$576
Blood cost savings		$745	$2,384–$3,576
Net cost savings^**c**^		$252	$1,891–$3,083

*FFP* fresh frozen plasma, *PLTs* platelets, *RBCs* red blood cells, *RBHS* radiofrequency bipolar hemostatic sealer. ^a^Calculated as $220/RBC unit, $50/FFP unit, and $600/PLT unit, the average cost of blood components in the mid-Atlantic region. ^b^Calculated as 3.2 to 4.8 times acquisition cost [[4]]. ^**c**^Calculated as blood cost savings minus RBHS device costs ($493).

## Discussion

For patients undergoing multilevel spine fusion surgery, the use of RBHS to achieve hemostasis significantly reduced blood loss, transfusion requirements, the decrease in Hb, and cost. These findings demonstrate the effectiveness of RBHS for blood conservation in patients undergoing these complex surgeries. Average blood loss was approximately 40% of total blood volume in the control group but less than 20% of total blood volume in the treatment group. Although various methods of blood conservation have been described for orthopedic and spine surgery (controlled hypotension [[7]], autologous blood salvage (cell saver) [[8],[9]], autologous intraoperative normovolemic hemodilution [[18]], and antifibrinolytic medications [[10]]), little published evidence has described the beneficial effects of new methods of electrocautery for achieving hemostasis. It is noteworthy that in addition to reducing blood loss and transfusion, the use of RBHS also reduced the variation in bleeding and transfusion to as little as one-third to one-half that in the control group, as assessed by standard deviation of the means for these factors. This finding suggests that RBHS may be useful in reducing blood loss for spinal fusion patients predisposed to the greatest amount of bleeding.

Traditional unipolar cautery produces hemostasis by burning tissue at temperatures up to 400°C [[12]]. In contrast, the RBHS method of cautery uses saline-cooled bipolar delivery of radiofrequency energy, which contracts and seals small blood vessels [[19]] at tissue temperature <100°C [[14],[20],[21]]. Some evidence also supports the efficacy of RBHS for hemostasis during bleeding from bone surfaces, by way of shrinking collagen in the walls of blood vessels [[22]]. Because the temperature is reduced by saline irrigation, RBHS causes less charring of the tissues and less injury to neural tissues [[11]], an important factor during spinal surgery. Advantages of achieving better hemostasis during spine surgery are not limited to reducing transfusion requirements. Better visualization of the delicate neural structures may result in better surgical outcomes; however, assessing this outcome would require a larger sample size, as neurologic injury is a relatively uncommon event.

Previous studies in total joint replacement [[14],[23]], liver resection [[13]], and scoliosis surgery [[16]] have reported reductions in bleeding and transfusion by amounts similar to those determined in our study. In the one study that showed no difference in blood loss or transfusion requirements with the use of RBHS in patients undergoing total hip arthroplasty, the RBHS device was used alone, without concomitant use of unipolar cautery, and the overall transfusion rates were very low (4%–6%) [[23]]. In our study, transfusion rates were high, owing to the complex spine procedures, and both types of cautery were used; our surgeons agree that for these procedures, both are needed. These findings suggest that RBHS may not be an adequate substitute for unipolar cautery during highly complex surgical procedures.

In addition to confirming the effectiveness of RBHS in spinal fusion surgery, we showed that patients have a substantially higher hemoglobin level at discharge when RBHS is used. Although the difference in morbidity outcomes and length of stay in our study were not statistically significant between groups, our study was not powered with a large enough sample size to adequately compare these outcomes. However, we did see a trend toward more favorable overall outcomes in the RBHS group. Considering the known association between allogeneic blood transfusion and increased morbidity and length of stay [[1]–[3]], it is conceivable that with a larger sample size, improved outcomes may become apparent when RBHS is used.

The use of RBHS for these complex spinal procedures led to significant cost savings because of the reduction in transfusion requirements. The method of calculating cost savings is clear when blood product acquisition costs are utilized. The activity-based costs, however, account for the many steps in the process of bringing blood all the way from the donor to the recipient. Admittedly, this method inflates the cost of blood, but each step does indeed add incremental cost at some level for management of the blood supply [[4]].

Our study had several limitations that should be recognized. First, the study design was retrospective in that it used a historical control group. To guard against bias, however, we chose consecutive patients for whom the RBHS device was used and propensity matched these patients to control patients based on clinical predictors of blood loss and transfusion. The patient characteristics (Table 1) illustrate that the two groups were comparable. Second, one particular outcome that we report, intraoperative estimated blood loss, is known to be a relatively inaccurate measure of actual bleeding [[24]] for a variety of reasons (irrigation in the suction container, blood on sponges, and blood on the floor and surgical drapes). However, because of the retrospective nature of the study, the clinicians who estimated blood loss were unaware of and uninvolved with the study and thus less likely to introduce bias in measuring this parameter. A particular strength in our study was the use of two comprehensive databases that have been previously validated [[6],[17]] to obtain both intraoperative and whole-hospital-stay blood utilization data. Previous studies focused primarily on intraoperative blood loss and transfusion alone [[16],[25]]. Our databases include whole hospital (from admission to discharge) nadir and discharge hemoglobin levels and utilization of both RBC and non-RBC blood components (FFP and PLTs).

## Conclusions

For patients undergoing multilevel lumbar, thoracic, or thoracolumbar spinal fusion surgery, the use of RBHS to achieve hemostasis results in reduced blood loss, reduced intraoperative and whole hospitalization transfusion requirements, higher discharge hemoglobin levels, and substantial cost savings. These findings suggest that using this new method as part of a comprehensive patient blood management program is an efficacious strategy.

## Abbreviations

FFP: Fresh frozen plasma

Hb: Hemoglobin

ORMIS: Operating room medical information system

PLTS: Platelets

RBC: Red blood cells

RBHS: Radiofrequency bipolar hemostatic sealer

## Competing interests

Steven M. Frank, MD, has received honoraria for speaking at educational seminars from the following companies: CSL Behring, Haemonetics, and Medtronic. These companies are all involved with patient blood management. Zia L. Gokaslan holds stock in Spinal Kinetics and U.S. Spine and received grants and research funds from DuPuy, AO North America, Integra, NREF, and Medtronic and honoraria and fellowship support from AO Spine North America. Khaled M. Kebaish has consulted for K2M and DePuy Spine, has had speaking/teaching arrangements with K2M and DePuy Spine, and has received research support (investigator salary) from K2M (paid directly to institution/employer) and DePuy Spine (paid directly to institution/employer).

## Authors’ contributions

SF was involved with design of the study, statistical analysis, and with manuscript preparation. JW was involved with the data interpretation, statistical analysis, and manuscript preparation. ID assisted with data collection and analysis. ZG, PN, and KK assisted with data interpretation and manuscript preparation. All authors read and approved the final manuscript.

## Authors’ information

SF is the Medical Director of the Patient Blood Management Program at Johns Hopkins Hospital. ZG is the Chief of Spine Surgery at Johns Hopkins Hospital. PM is the Director of Transfusion Medicine at the Johns Hopkins Hospital.
